# Is the Imitative Competence an Asymmetrically Distributed Function?

**DOI:** 10.3389/fnsys.2021.791520

**Published:** 2021-12-24

**Authors:** Mara Fabri, Chiara Pierpaoli, Nicoletta Foschi, Gabriele Polonara

**Affiliations:** ^1^Department of Life and Environmental Sciences, Marche Polytechnic University, Ancona, Italy; ^2^UOST, INPS Casilino-Prenestino, Rome, Italy; ^3^Neurological Clinic, Epilepsy Centre, Ancona University Hospital Umberto I, Ancona, Italy; ^4^Department of Odontostomatologic and Specialized Clinical Sciences, Marche Polytechnic University, Ancona, Italy

**Keywords:** imitation, anatomical perspective, intransitive gestures, mental rotation, cortical activation, corpus callosum, split-brain

## Abstract

This study reconsiders behavioral and functional data from studies investigating the anatomical imitation (AI) and the related mental rotation (MR) competence, carried out by our group in healthy subjects, with intact interhemispheric connections, and in split-brain patients, completely or partially lacking callosal connections. The results strongly point to the conclusion that AI and MR competence requires interhemispheric communication, mainly occurring through the corpus callosum, which is the largest white matter structure in the human brain. The results are discussed in light of previous studies and of future implications.

## Introduction

This study described imitative behavior and its relationships with mental rotation (MR). Imitative behavior is a form of interaction emerging in the first month of life, allowing babies to do what they see adults doing. Imitation is to copy spontaneously or on education what another individual acts, or to reproduce the behavior of another individual serving as a model (Mühlau et al., 2005). Since the late 1970s, studies on imitation suggested that few-hour-old babies are able to imitate facial expressions (i.e., tongue protrusion, lips and mouth opening), demonstrating that a tendency to imitate is present from birth (Meltzoff and Decety, 2003; Meltzoff, 2007).

In the past, several aspects of imitation have been addressed [see literature in Pierpaoli et al. (2018, 2020a)] with respect to (1) type of stimulus (hand/head vs. finger configurations; novel vs. known actions; transitive vs. intransitive gestures; biological vs. non-biological movements; object-oriented vs. not oriented movements); (2) the spatial position of stimulus and observer, e.g., degrees of rotation at 0° and 180°; (3) instructions, e.g., anatomical perspective vs. mirror; (4) a combination of the previous factors.

It is known that in particular situations, the imitative competence is impaired, as in children with autism spectrum disorders (Rogers et al., 2003); in these patients, the lack of socio-communicative abilities would be secondary compared with altered development of skills such as imitation. Inability to MR performance is also reported in various pathological situations showing compromised imitation, such as schizophrenia, autism, dementia, and other diseases related to cognitive deficits (Hardan, 2000; Paul, 2011).

Many clinical and experimental studies showed that in psychiatric and neurological disorders in which the imitative competence and MR are reduced, morphological structural alterations of the corpus callosum (CC) are also evident, in shape and/or size and/or myelination (Hardan, 2000; Paul, 2011; Fujino et al., 2014). It could be thus hypothesized a causal link between good MR ability and anatomical imitation (AI) and the integrity of the CC.

This study describes our recent researches focused on the anatomical perspective-taking in imitating intransitive meaningful gestures, on the MR ability, and on their relationships with the CC. Since AI is basic for learning, for social behavior, and for rehabilitative therapies, the knowledge of the neural circuits underpinning AI and MR and of the timing of CC development and maturation could have important influences on the children and adolescent educational and social integration programs and on physical therapy programs.

## Imitation

The imitation is a behavior reproducing observed actions; it allows individuals to establish the first form of relationship between infants and parental figures, and it is basic for learning. In everyday life, the actions to be imitated are generally presented with the imitator facing the model, i.e., in third-person perspective.

When invited to imitate gestures of someone facing them, individuals can choose either an anatomical mode, activating exactly the same effectors as the model (therefore the same nervous mechanisms), or a specular (mirror) mode, activating the effectors sharing an external spatial reference with those activated by the model (Koski et al., 2003; Franz et al., 2007; Press et al., 2009).

Previous investigations on imitative competence showed that (1) young children were likely to use a mirror mode imitation strategy, but with increasing age, the anatomical perspective prevailed (Wapner and Cirillo, 1968); (2) performance was more accurate when participants imitated with the opposite body part facing model at 180° and 240° (Press et al., 2009); adult subjects made significantly fewer errors when using the mirror mode (“imitate as if looking at a mirror”) than when asked to use the opposite hand (anatomical correspondence; Avikainen et al., 2003).

Therefore, it was hypothesized that the natural tendency of humans is to mirror movements, and anatomical performances would replace the mirror in the presence of certain stimulus information (Franz et al., 2007). When the mental alignment between the self and the stimulus does not need mental spatial transformation, the performance is faster and occurs in the mirror mode; when a rotation of the body representation of an individual is necessary to align with respect to the stimulus, i.e., a spatial transformation is necessary, the anatomical performance would be the result.

The choice to mirror acts executed by others could be likely related to cortical patterns of activity, consistent with the existence of the mirror neuron system (MNS), and reflecting a close connection between the mirror strategy and the specific neuron system that matches observed and executed actions.

Investigations of cortical activation during anatomically and not anatomically matching gestures have highlighted different functional MRI patterns of frontoparietal activation for mirror and AI (Koski et al., 2003), generating the hypothesis of a close link between mirror imitation and MNS. In addition, since the anatomical perspective is more often associated with executive errors (Ishikura and Inomata, 1995) and the mirror imitation involves shorter reaction times than the anatomical one (Koski et al., 2003; Franz et al., 2007), it can be hypothesized that the two perspective-takings may be subtended by distinct neural processes.

### Behavioral Studies

Recent behavioral studies from our group tested the hypothesis of the mirror mode of imitation as the primary one. For this purpose, adult healthy participants (Pierpaoli et al., 2014) and epileptic patients who undergone callosal resection to prevent the spread of seizures (callosotomized patients; Pierpaoli et al., 2018) were invited to imitate intransitive meaningful gestures, performed by a human model shown in a video. In the first condition, the subjects were asked to imitate freely (free imitation); in the second condition, the subjects had to use the same or opposite body part (driven imitation).

The investigation was focused on the perspective assumed by the subjects in imitating gestures, to evaluate whether the participants’ choice preferred a mirror-mode or an anatomical perspective-taking.

To perform the driven imitation task, the subject has to primarily conceptualize the term *same*. The *same* with respect to what/whom? Encoding the concepts of *same* requires the analysis of spatial coordinates; someone could choose a purely spatial matching between the stimulus and the response (like to be in front of a mirror) or could change its mental body image (through an abstract operation as the MR) so as to align one’s body parts with the other’s body parts.

In the above-mentioned studies, participants’ responses produced enlightening results: both groups used the mirror perspective when free to imitate, but only the control group (with intact CC) used the anatomical perspective when asked to use the *same* limb. This lays for split-brain patients to differently encode for the *same* concept, or not to be able to translate the *same* concept into a motor schema based on anatomical criteria.

### Functional Studies

Later, the cortical activation pattern evoked in the two previous conditions, i.e., mirror and AI of intransitive gestures, was investigated with functional MRI (fMRI). To reduce artifacts, distortions, and signal loss, some adaptation was made to the experimental protocol: in the free imitation task, participants had to simply watch the videos, by assuming that simple gesture observation does activate the cortical pattern for mirror imitation; in the driven imitation session, they had to image to imitate gestures using the same limb as the model.

This study was aimed to identify the following in healthy control subjects:

1. The cortical areas activated during the observation of intransitive meaningful gestures performed by a model, presented in video clips in 3rd person perspective (condition likely simulating the mirror mode imitation).
2. The cortical areas activated by imaging to imitate intransitive meaningful gestures performed by a 3rd person model, using the same limb of the model (condition likely simulating the anatomical mode imitation).

**FIGURE 1:**
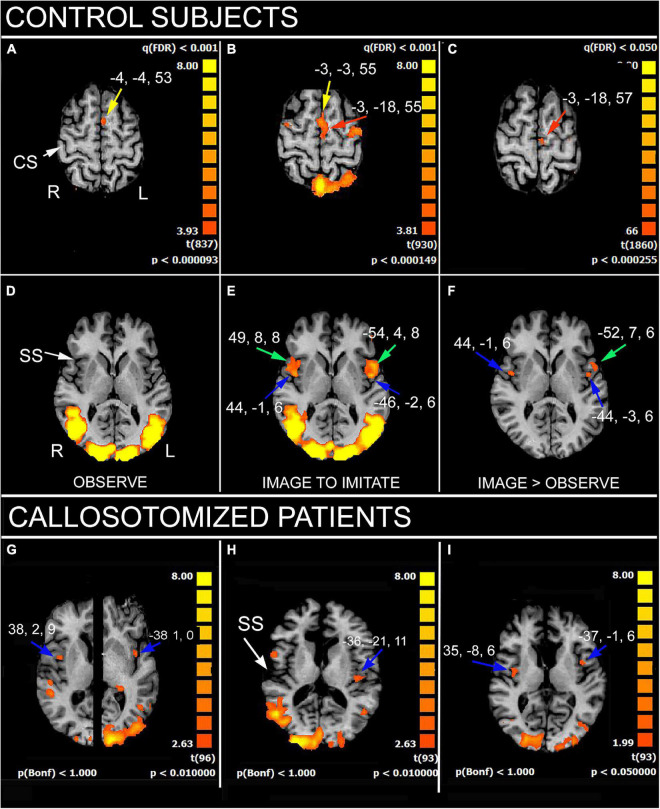
Significant activation in imitation task, in control subjects **(A–F)**, as obtained from multisubjects analysis, and in three callosotomized patients **(G–I)**. **(A,D)** OBSERVE condition: activation of anterior left supplementary motor area (SMA; **A**, yellow arrows) is evident. *z* values in **(A,D)** are 53 and 6, respectively. **(B,E)** IMAGE TO IMITATE condition: activation of anterior (**B**, yellow arrow) and posterior left SMA (**B**, red arrow) is shown. Bilateral activation in IFG (area 44; **(E)**, green arrows) and opercular cortex (**E**, blue arrows) is also visible. *z* values in **(B,D)** are 55 and 6, respectively. **(C,F)** IMAGE TO IMITATE>OBSERVE: only the activation in left posterior SMA (**C**, red arrow), left IFG (**F**, green arrow), and bilateral parietal opercula (**F**, blue arrows) is evident. *z* values are 57 and 6, in **(C,F)**, respectively. **(G–I)** IMAGE TO IMITATE condition in three patients: in **(G)** (total callosotomy) and **(I)** (anterior callosotomy) bilateral activation foci in opercular cortex are evident (blue arrows); in **(H)** (total callosotomy) in left hemisphere only. Axial images in **(H,I)** are from the same *z* values; in **(G)**, the two hemispheres are from different *z* values because of different position of the opercular activation foci. CS, central sulcus; SS, Sylvian sulcus; according to the radiological convention, the left hemisphere is shown on the right. Modified from Pierpaoli et al. (2020a,2021a).

The results showed that, in control subjects, the simple gestures’ observation activated the cortical regions belonging to the MNS (Pierpaoli et al., 2020a): left medial area 6, bilateral motor cortex in the precentral gyrus (PrG; area 4), left inferior parietal lobule (IPL), and bilateral angular gyrus (Figure 1); all these areas built a frontoparietal network known as the observation-execution matching system, having the role to recognize the action (Rizzolatti et al., 2014; Rizzolatti and Sinigaglia, 2016), and to be the neural substrate underlying the action comprehension and eventual imitation. The imaging condition evoked a more extensive activation in the right temporoparietal junction (TPJ) including a larger portion of area 39; in addition, a bilateral activation in the medial (MFG) and inferior frontal gyri (IFG) was observed. Activation also appeared in the left area 45 and was bilateral in area 44 and in the parietal opercula (PO).

A similar activation pattern was observed in patients in the first task: the activation of the MNS cortical circuitry evoked by sole observation was, however, less consistent than in control subjects (Pierpaoli et al., 2021a). A different activation pattern was instead elicited in the second task, image to imitate with the same limb: the bilateral activation of PO was observed only in two patients, one of whom did perform AI (Figure 1). This suggested that a good interhemispheric connection, other than a certain pattern of cortical activation, is necessary to perform AI (Pierpaoli et al., 2021a).

In fact, our results indicate that the imitation according to an anatomical criterion seems to require the cooperation of cortical areas in both hemispheres: the MFG, IPL, and IFG in the left, the TPJ in the right, and the PO in both hemispheres (see also Caspers et al., 2010).

Therefore, the behavioral and functional results described above have given the input to design further research concerning the mechanism of MR, its involvement in the perspective assumption in imitation behavior, and the role of the CC in this mental operation.

## Mental Rotation

Mental rotation is an abstract operation whereby a person imagines rotating an object or a body part to place it in a different position. MR was first studied in behavioral experiments, showing that the time to make a judgment about a rotated object increases in a near-linear fashion with the amount of rotation required to align the object with a comparison one or with a previously learned template (Shepard and Metzler, 1971; Cooper and Shepard, 1973; Zacks, 2008). This effect has been observed with different kinds of stimuli: geometric and “abstract,” such as letters, lines, polygons, and three-dimensional cubes, and embodied and concrete, such as hands, legs, and whole-body figures (Cooper and Shepard, 1975; Kosslyn, 1988; Parsons, 1994; De Lange et al., 2006). The MR ability is present in very young children, reaches higher levels during adolescence, and declines with aging [see data and literature in Iachini et al. (2019)], with the severity of decline often depending on stimulus and task type (object-based vs. egocentric; Jansen and Kaltner, 2014).

Previous studies, trying to allocate this abstract function in the brain, suggested that objects’ MR belongs to the right hemisphere and body images’ MR to the left, although with less evidence (Parsons, 2003; Tomasino et al., 2004; Zacks, 2008); however, others studies go against the right hemisphere dominance for objects MR (Corballis, 1997; Serrati et al., 2000).

Mental rotation is strictly concerned with AI, as suggested in previous behavioral studies. It was therefore hypothesized that the different AI performances of the split-brain patients with respect to controls could possibly be due to an impaired capacity for MR, in which the CC might have a role. Assuming the existence of a causal link between callosal fiber functionality and the AI performance, the MR ability was investigated in the same group of subjects previously tested, i.e., patients with partial or total resections of the CC, and healthy adults with intact CC.

### Behavioral Studies

To test the hypothesis of a central role of the interhemispheric connections in MR, two separate experimental sessions were set: a verbal task, in which participants answered by voice whether the hand holding the cup in the displayed picture was the left or right, and a motor task, where participants responded by lifting their own left or right hand. In this task, it was possible to evaluate the MR ability through a hand-laterality judgment.

The results demonstrated that control subjects performed MR almost perfectly, in verbal and in motor sessions, both with the model in first and in third-person perspective. Callosotomized patients showed some impairment, mainly in the verbal session and when the model was in 3rd person. The results indicated the central role of interhemispheric connections in MR, and therefore, because of the need for the cooperation of both hemispheres to be performed, strongly suggest considering the MR as an asymmetric function (Pierpaoli et al., 2020b).

The behavioral data just described all agree in the observation that, concerning egocentric transformation, back orientation (first-person presentation) produces better performances than front orientation (third-person presentation). The difference increases in childhood and senior age, both periods of life in which alterations of the cerebral white matter are observed.

### Functional Studies

**FIGURE 2:**
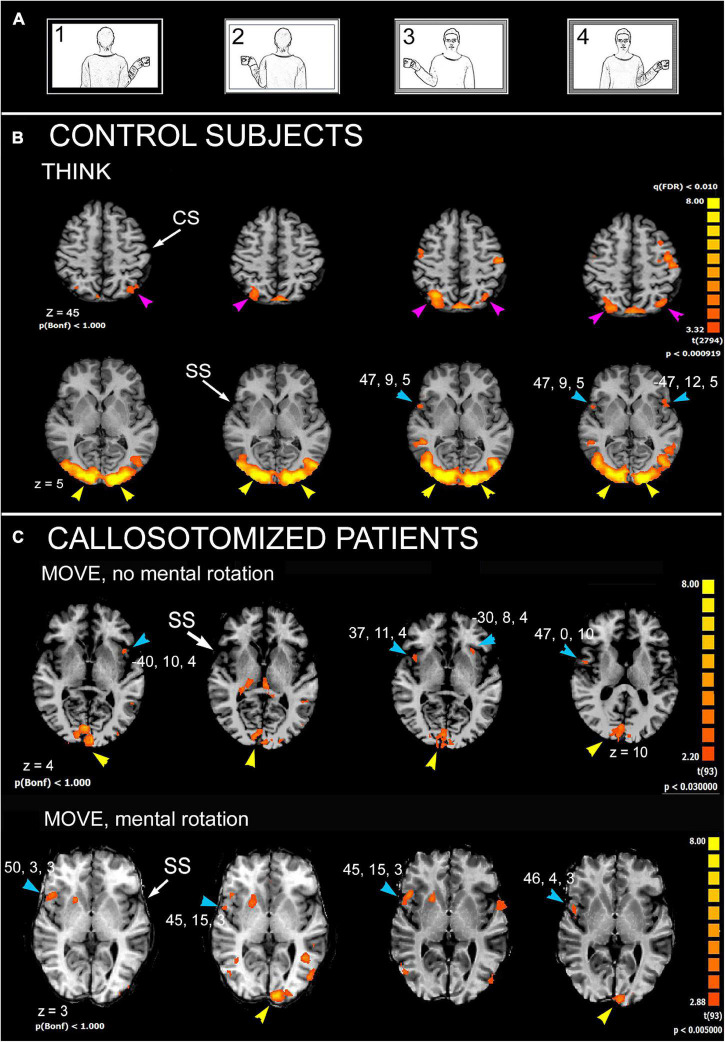
Significant activation in mental rotation (MR) task, in control subjects, as obtained from the multisubject analysis, and in two callosotomized patients. **(A)** Stimulation protocol. **(B)** Control subjects, THINK condition. Axial images are from *z* = 5 and *z* = 45. **(C)** Callosotomized patients, MOVE condition: activation from two patients are shown: the first did not perform mental rotation (top row), the second did (bottom row). CS, central sulcus; SS, Sylvian sulcus; according to the radiological convention, the left hemisphere is shown on the right. Modified from Pierpaoli et al. (2021b).

Subsequent research was designed with the aim to identify, by fMRI, the cortical areas activated during an MR task with human body pictures. Attention was also paid to relate the extent of callosal resection with MR ability. A block-designed protocol derived from that previously used in the behavioral study (Pierpaoli et al., 2020b) was administered. The results indicated that regions involved in MR in control subjects include lateral area 6 of PrG and area 7 of the superior parietal lobule (SPL) in both hemispheres; area 22 of STG (TPJ) in the right hemisphere and area 13 of PO in both (Figure 2). In callosotomized patients, the activation pattern was similar (Figure 2); however, at variance with control subjects, the activated areas were less consistently found in either hemisphere, and the presence of unilateral or bilateral activation was independent of the ability to perform MR. The hypothesis is that due to a total or partial lack of the CC, callosotomized subjects are not able to integrate information between the hemispheres, and therefore inefficient to perform MR when the model was in third-person perspective.

## Discussion

The results from imitation studies provide further support to the notion that the AI requires the cooperation of both hemispheres, since different cortical areas were activated in different hemispheres: posterior area 22 and 39 (TPJ) in the right, dorsal premotor area 6 (MGF), areas 39–40 (IPL), and area 44 (IFG) of the left, and the PO of both.

Previous functional studies, revised by Lesourd et al. (2018), also found activation in the insula, especially the right one. Indeed, it has been proposed that the insular cortex may play a critical role in self-awareness of limb movement and sense of limb ownership (Karnath and Baier, 2010). Evidence from patients with brain damage reports a disturbing sense of agency or of limb ownership, more frequently after right hemisphere lesions (Baier and Karnath, 2005). Thus, the right insula could be a central node of the network involved in human body schema representation; it can therefore be assumed that to imitate someone else, a person must integrate signals from various parts of his/her own body and distinguish them from body parts of the model to imitate. It has been shown that IPL is also involved in this function (Decety and Sommerville, 2003; Tessari et al., 2021), but a side preference has still to be defined.

The cortical areas activated during the AI task communicate with their homologs in the contralateral hemisphere through the CC: area 6 by sending fibers in the central portion (Chao et al., 2009), area 44 in the ventral rostral body and ventral anterior midbody, IPL across the dorsal splenium, and TPJ the ventral splenium (Chao et al., 2009). The interhemispheric fibers connecting the PO of the two sides seem to travel through the anterior and central callosal body (Fabri et al., 1999, 2001, 2006, 2011; Polonara et al., 2014; Mascioli et al., 2015). Accordingly, patients lacking callosal fibers from the anterior and/or central body could display an impairment in AI performance, although the involved cortical areas are generally activated.

The involvement of the CC has also been suggested in a previous study reporting a gradual shift toward the AI mode in children, as the maturation of the CC progress with age, from 8 to 18 years (Wapner and Cirillo, 1968). In addition, by assuming that AI does require the abstract operation of MR, present results are also in line with previous behavioral data on the same patients (Pierpaoli et al., 2020b), demonstrating that callosotomized patients perform worse than intact brain subjects in an MR task.

These results from MR investigation are essentially in line with previous numerous studies [refer to data and literature in Milivojevic et al. (2009)], indicating that regions participating in MR include lateral areas 6 of PrG, 7 of SPL, 13 of PO in both hemispheres, and 22 of STG (TPJ) in the right. Previous functional studies (Zacks, 2008; Thirioux et al., 2010; Tomasino and Gremese, 2016) identified a common trend correlating MR with a sort of hemispheric specialization: left lateralization for the parietal cortex and a right specialization for the frontal regions. Similar activation was found, although less consistently, also in callosotomized patients, both in those performing MR and in those not. In particular, in all patients, the activation of PO was reported, at least on the right side. This observation strongly points to the role of the PO both in AI and in MR.

## Conclusion

Since one big difference between control subjects and patients is the total or partial lack of the CC, it can be concluded that both AI and MR require the integrity of the CC and, therefore, the imitative competence is an asymmetrically distributed function. In this study, the findings described confirms that people with partial or total callosal resection display reduced performance in laterality test with stimuli in third person orientation, suggesting an alteration of MR mechanism (Pierpaoli et al., 2020b); consequently, the ability to select AI is also compromised. In addition, it appears that the CC is involved in a cognitive task. However, more studies are necessary to obtain further insight on this function of the CC and to investigate the role of the right insula in imitation.

Future studies could collect similar data from healthy children and adolescents of different ages and from young and adult people with defective neural development or neural lesions. Since AI and MR are basic for learning, social behavior, and rehabilitation, the comprehension of the neural circuit underpinning them and the timing of the CC development could be helpful to set efficient programs both for children and adolescents educational and social integration and for physical therapy.

## Data Availability Statement

The data analyzed in this study is subject to the following restrictions: Privacy. Requests to access these datasets should be directed to MF. Data are available with the permission of Ancona University Hospital Umberto I.

## Ethics Statement

The studies involving human participants were reviewed and approved by Comitato Etico Regionale Marche. All subjects involved provided their written informed consent to participate in this study.

## Author Contributions

MF and CP equally contributed to the conception, design of the study and data analysis and interpretation. NF, CP, and GP contributed to data collection. MF prepared the draft article that was critically revised by GP and CP. All authors contributed to the article and approved the submitted version.

## Conflict of Interest

The authors declare that the research was conducted in the absence of any commercial or financial relationships that could be construed as a potential conflict of interest.

## Publisher’s Note

All claims expressed in this article are solely those of the authors and do not necessarily represent those of their affiliated organizations, or those of the publisher, the editors and the reviewers. Any product that may be evaluated in this article, or claim that may be made by its manufacturer, is not guaranteed or endorsed by the publisher.
